# Predictors of Viral Pneumonia in Patients with Community-Acquired Pneumonia

**DOI:** 10.1371/journal.pone.0114710

**Published:** 2014-12-22

**Authors:** Ji Eun Kim, Uh Jin Kim, Hee Kyung Kim, Soo Kyung Cho, Joon Hwan An, Seung-Ji Kang, Kyung-Hwa Park, Sook-In Jung, Hee-Chang Jang

**Affiliations:** Department of Internal Medicine, Chonnam National University Medical School, Gwang-ju, Republic of Korea; University of Dundee, United Kingdom

## Abstract

**Background:**

Viruses are increasingly recognized as major causes of community-acquired pneumonia (CAP). Few studies have investigated the clinical predictors of viral pneumonia, and the results have been inconsistent. In this study, the clinical predictors of viral pneumonia were investigated in terms of their utility as indicators for viral pneumonia in patients with CAP.

**Methods:**

Adult patients (≥18 years old) with CAP, tested by polymerase chain reaction (PCR) for respiratory virus, at two teaching hospitals between October 2010 and May 2013, were identified retrospectively. Demographic and clinical data were collected by reviewing the hospital electronic medical records.

**Results:**

During the study period, 456 patients with CAP were identified who met the definition, and 327 (72%) patients were tested using the respiratory virus PCR detection test. Viral pneumonia (n = 60) was associated with rhinorrhea, a higher lymphocyte fraction in the white blood cells, lower serum creatinine and ground-glass opacity (GGO) in radiology results, compared to non-viral pneumonia (n = 250) (p<0.05, each). In a multivariate analysis, rhinorrhea (Odd ratio (OR) 3.52; 95% Confidence interval (CI), 1.58–7.87) and GGO (OR 4.68; 95% CI, 2.48–8.89) were revealed as independent risk factors for viral pneumonia in patients with CAP. The sensitivity, specificity, positive- and negative-predictive values (PPV and NPV) of rhinorrhea were 22, 91, 36 and 83%: the sensitivity, specificity, PPV and NPV of GGO were and 43, 84, 40 and 86%, respectively.

**Conclusion:**

Symptom of rhinorrhea and GGO predicted viral pneumonia in patients with CAP. The high specificity of rhinorrhea and GGO suggested that these could be useful indicators for empirical antiviral therapy.

## Introduction

CAP remains a significant cause of morbidity and mortality [1], [2]. The development and application of diagnostic tests with improved sensitivity, such as the polymerase chain reaction (PCR), have led to recognition of the increasing role of respiratory viruses in CAP in all age groups [3]. These common respiratory viruses include influenza, parainfluenza viruses, adenoviruses, coronaviruses, respiratory syncytial viruses (RSV), metapneumoviruses and bocaviruses [4]–[6]. Evidence of viral infection was detected in 22% of CAP in adults [7]. Moreover, viruses were frequently found in the airways of patients requiring admission to intensive care units (ICU) with pneumonia, and patients with viral and bacterial infections had comparable mortality rates [7]–[11].

There are a number of studies on the subject of antiviral treatment for viral infections. Several studies showed the efficacy of antiviral agents including oseltamivir, zanamivir, amantadine and ribavirin [10], [12]–[17]. But, the Cochrane review of randomized controlled trials of antiviral agents does not demonstrate efficacy in the treatment of influenza [18]. However, the original studies included in the Cochrane review did not include people with severe underlying disorders or patients with a severe presentation of influenza. For this reason, no conclusion can be made on the efficacy of antiviral treatment for viral pneumonia by the Cochrane review [19]. There is evidence of efficacy in the treatment of influenza pneumonia [20]–[22], and early empirical antiviral therapy is still recommended in critically ill patients in whom viral pneumonia is suspected [7].

Although viral pneumonia is increasingly recognized as a major cause of CAP and early antiviral therapy can reduce mortality, few studies have investigated the clinical predictors of viral pneumonia, and the results have been inconsistent [23]–[26]. Moreover, evaluations of the diagnostic value of any clinical parameters, including sensitivity, specificity, and positive and negative predictive values, have not been performed. Although PCR methods are sensitive and real-time PCR enables rapid results in a clinically relevant time period, use of PCR is sometimes limited in CAP patients due to the associated costs [27]. This highlights the need for clinical predictors of viral infections in patients with CAP.

In this study, we describe the clinical parameters of viral pneumonia that would be useful in the development of diagnostic tests for respiratory viruses and early empirical antiviral treatment in patients with CAP.

## Patients and Methods

### Ethics statement

This study was approved by the Institutional Review Board of Chonnam National University Hospital. A waiver of the requirement for consent was granted given the retrospective nature of the project.

### Patients

Adult patients (≥18 years old) with CAP, who had tested for respiratory viruses by PCR in hospitalized patients and out-patients at Chonnam National University Hospital (900 beds, Gwang-ju, Republic of Korea) and Chonnam National University Hwasun Hospital (600 beds, Hwasun, Republic of Korea) between October 2010 and May 2013, were retrospectively identified. A case report form (CRF) was recorded at the time of admission for all pneumonia patients, which included clinical symptoms, underlying diseases, vital signs, CURB-65 score (the confusion, urea, respiratory rate, blood pressure, and aged 65 years or over score), and score on the pneumonia severity index (PSI). We reviewed the CRF which was stored in the hospital's electronic medical records.

### Definition

Pneumonia was defined as an acute illness with radiographic pulmonary infiltration, with at least one of the following being present: fever >38°C, WBC >12,000/mm^3^ or <6,000/mm^3^, and change in the mental status in elderly patients over the age of 70 years [24]. CAP is defined as pneumonia acquired outside a hospital or long-term care facility. It occurs within at least 48 hours of hospital admission or in a patient presenting with pneumonia who does not have any of the characteristics of health care-associated pneumonia (i.e., hospitalized in an acute care hospital for two or more days within 90 days of infection; residing in a nursing home or long-term care facility having received recent intravenous antibiotic therapy; undergoing chemotherapy; chronic dialysis within the past 30 days; or wound care within the past 30 days of the current infection). The exclusion criteria were solid organ transplantation and anyone with a diagnosis of active tuberculosis or a fungal infection [28]. In this study, the viral pneumonia was confirmed by respiratory viruses multiplex PCR using respiratory specimens of the patients. Classification of radiologic findings was three types by chest computerized tomography (CT) confirmed by one clinician and two radiologists; lobar consolidation, centrilobular nodule, GGO.

Viral pneumonia was diagnosed using the Influenza Antigen Rapid Test kit (SD BIOLINE, Young-in, Republic of Korea), the Anyplex II RV16 detection kit (Seegene, Seoul, Republic of Korea), or a multiplex virus RT-PCR. Viruses that can be diagnosed using the above equipment include influenza viruses A and B; adenoviruses; RSV A and B; parainfluenza viruses 1, 2, and 3; coronaviruses 229E, NL63, and OC43; metapneumoviruses; rhinoviruses; enteroviruses; and bocaviruses. This test is generally done using specimens from nasopharyngeal/oropharyngeal swabs, sputum, or transtracheal aspirate. The sensitivity and the specificity of the multiplex PCR kit used in this study are reported to be 95.2% and 98.6% [29].

Pneumococcal pneumonia was diagnosed when *Streptococcus pneumoniae* was isolated from a normally sterile blood or sputum, or the urine antigen assay for *S. pneumoniae* (Alere BinaxNOW, Young-in, Korea) was positive. Microbiological sampling was taken at the time of admission before antimicrobial treatment commenced.

### Statistical analyses

Categorical variables were compared using Fisher's exact test or the Pearson χ2 test, and continuous variables were compared using Student's t-test or Mann-Whitney U-test, as appropriate. Multivariate analyses were performed using the logistic regression model in the backward stepwise conditional manner.

All significance tests were two-tailed, and *p-*values ≤0.05 were deemed to indicate statistical significance. Statistical analyses were performed using the SPSS software version 21.0 (IBM Corporation, Armonk, NY, USA).

## Results

### Etiology of CAP

During the study period, 456 patients with CAP were identified who met the definition, and 327 (72%) patients were tested with the respiratory virus PCR. Among the 327 patients, an etiologic diagnosis could be established in 204 cases (62%). In this study, 317 patients were tested using specimens from nasopharyngeal swabs or sputum, with the exception of 10 patients who were intubated at admission using specimens from transtracheal aspirate.

Among 327 patients, respiratory viruses were detected in 60 (18%) patients, while 250 (76%) patients were diagnosed with non-viral pneumonia with negative PCR results, and 17 (5%) patients were diagnosed as being co-infected with a virus and bacteria. Co-infected patients (n = 17, 5%) were excluded from the study.

Among the 60 patients with respiratory viruses, influenza viruses was most common (n = 23, 38%). Other etiological agents included RSV (n = 9, 15%), rhinoviruses (n = 7, 12%), coronaviruses (n = 6, 10%), adenoviruses (n = 5, 8%), metapneumoviruses (n = 5, 8%), parainfluenza viruses (n = 3. 5%) and bocaviruses (n = 2, 3%).

Among 250 cases of non-viral pneumonia, *Streptococcus pneumoniae* pathogen was most common (n = 88, 35%). Other etiological pathogen included *Mycoplasma pneumoniae* (n = 10, 4%), *Klebsiella pneumoniae* (n = 7, 3%), *Staphylococcus aureus* (n = 5, 2%), *Haemophilus influenza* (n = 5, 2%), *Escherichia coli* (n = 3, 1%), *Pseudomonas aeruginosa* (n = 2, 1%), *Moraxella catarrhalis* (n = 1, <1%), *Proteus mirabilis* (n = 1, <1%) and *Peptostreptococcus* spp. (n = 5, 2%).

### Clinical features and outcomes of viral pneumonia compared to non-viral CAP

The clinical features and the outcomes of CAP are shown in Table 1. No differences were found in terms of age and gender ratio in a comparison of viral and non-viral pneumonia. No significant difference was found in co-morbidity between viral and non-viral pneumonia. Viral pneumonia was characterized by a higher frequency of rhinorrhea, compared to non-viral pneumonia (*p* = 0.012). There was no significant difference in other symptoms between viral and non-viral pneumonia.

**Table 1:** Clinical features and outcomes of 310 patients with viral or non-viral community-acquired pneumonia.

	No. (%) of patients		
Characteristics	Viral Pneumonia (N = 60)	Non-viral Pneumonia (N = 250)	*P* value
Demographic data			
Male sex	43 (72)	194 (78)	0.397
Age^a^	67 (±14)	70 (±11)	0.106
Underlying diseases			
Diabetes mellitus	12 (20)	50 (20)	>0.999
Hypertension	15 (25)	77 (31)	0.433
Cancer	14 (23)	45 (18)	0.362
Chronic obstructive lung disease	12 (20)	79 (32)	0.084
Ischemic heart disease	7 (12)	21 (8)	0.452
Cerebral vascular accident	2 (3)	14 (6)	0.746
Chronic kidney disease	1 (2)	13 (5)	0.319
Symptoms			
Fever	41 (68)	152 (61)	0.373
Cough	43 (72)	149 (60)	0.103
Sputum	31 (52)	134 (54)	0.886
Rhinorrhea	13 (22)	23 (9)	0.012
Dyspnea	22 (37)	112 (45)	0.310
Chest pain	3 (5)	26 (10)	0.228
Diarrhea	4 (7)	12 (5)	0.523
Severity and Outcomes			
CURB-65^b^	1.3 (1, 2)	1.4 (1, 2)	0.094
PSI^b^	97 (68, 116)	102 (73, 126)	0.188
ICU admission	8 (13)	44 (18)	0.564
Mechanical ventilation	10 (17)	30 (12)	0.390
30-day mortality	10/58 (17)	38/249 (15)	0.843
30-day attributable mortality	9/58 (16)	38/249 (15)	>0.999

Continuous variables were expressed as means ± SDs^a^ or medians (IQRs)^b^ and were compared by the Student's t test^a^ or Mann-Whitney U test^b^.

CURB-65: Confusion-Urea-Respiratory-Blood pressure-65 score, PSI: Pneumonia severity index, ICU: Intensive care unit.

In terms of severity, CURB-65, PSI, ICU admission, mechanical ventilation and 30-day mortality were not significantly different between the two groups.

All of the patients with CAP were treated with empirical antibiotics at admission. After they tested positive for viral PCR, the influenza virus was detected in 23 patients who were given the antiviral agent; oseltamivir (n = 17) or peramivir (n = 6).

### Laboratory and radiological findings of viral pneumonia compared to non-viral community-acquired pneumonia

The laboratory and radiological findings of CAP resulting from viruses or bacteria are shown in Table 2. Viral pneumonia was associated with a significantly higher lymphocyte fraction in the white blood cells, and significantly lower serum creatinine levels than non-viral pneumonia (*p*<0.05, each). However, no significant differences were found in the total white blood cell counts (WBC) and C-reactive protein (CRP).

**Table 2:** Laboratory and radiological findings of 310 cases of viral or non-viral community-acquired pneumonia.

	No. (%) of patients		
Characteristics	Viral Pneumonia (N = 60)	Non-viral Pneumonia (N = 250)	*P* value
Laboratory findings			
White blood cell counts (/mm^3^)^a^	12985 (7925, 12550)	13946 (9950, 17120)	0.300
Neuotrophil %^a^	77 (71, 86)	78 (76, 89)	0.239
Lymphocyte %^a^	13 (7, 18)	11 (5, 14)	0.032
C-reactive protein (mg/dL)^a^	14 (7, 20)	14 (5, 13)	0.764
Serum creatinine (mg/dL)^a^	1.1 (0.6, 1.3 )	1.4 (0.8, 1.5)	0.025
Radiologic findings			
Lobar consolidation	17 (28)	111 (44)	0.028
GGO	26 (43)	39 (16)	<0.001
Centrilobular	17 (28)	100 (40)	0.104

Continuous variables were expressed as medians (IQRs)^a^ and were compared by Mann-Whitney U test^a^. GGO: Ground-glass opacity.

All of the patients underwent a chest CT. The GGO radiology pattern on chest CT was more frequently observed in viral than non-viral pneumonia (*p*<0.01).

### Clinical features and outcomes, laboratory and radiological findings of influenza pneumonia compared to pneumococcal pneumonia

No differences were found in terms of age and gender ratio between influenza and pneumococcal pneumonia (Table 3). In terms of severity, the CURB-65 score, PSI, mechanical ventilation and 30-day mortality were not significantly different between the two groups (Table 3). However, influenza pneumonia was associated with significantly higher rates of rhinorrhea, and GGO on radiological findings than pneumococcal pneumonia (*p*<0.05, each).

**Table 3:** Clinical features, outcomes, laboratory and radiological findings of influenza pneumonia, compared to pneumococcal pneumonia.

	No. (%) of patients		
Characteristics	Viral Pneumonia (Influenza) (N = 23)	Pneumococcal Pneumonia (N = 88)	*P* value
Demographic data			
Male sex	19 (83)	73 (83)	>0.999
Age^a^	71 (±10)	69 (±11)	0.342
Underlying diseases			
Diabetes mellitus	8 (35)	18 (20)	0.161
Hypertension	7 (30)	24 (27)	0.793
Chronic obstructive lung disease	6 (26)	31 (35)	0.463
Cancer	7 (30)	14 (16)	0.140
Ischemic heart disease	3 (13)	12 (14)	>0.999
Symptoms			
Fever	17 (74)	46 (52)	0.097
Cough	17 (74)	51 (58)	0.231
Sputum	11 (48)	52 (59)	0.348
Rhinorrhea	6 (26)	6 (7)	0.017
Dyspnea	8 (35)	46 (52)	0.161
Severity and Outcomes			
CURB-65^b^	1.7 (1, 2)	1.5 (1, 2)	0.981
PSI^b^	117 (78, 136)	104 (75, 122)	0.286
Mechanical ventilation	6 (26)	13 (15)	0.526
30-day mortality	6 (26)	15 (17)	0.375
Laboratory findings			
White blood cell counts (/mm^3^)^b^	13070 (7800, 18400)	14410 (9425, 17275)	0.382
Neuotrophil % ^b^	76 (70, 87)	78 (76, 90)	0.240
Lymphocyte % ^b^	15 (7, 21)	11 (6, 15)	0.081
C-reactive protein (mg/dL)^b^	17 (8, 26)	13 (4, 18)	0.056
Serum creatinine (mg/dL)^b^	1.3 (0.7, 1.5)	1.6 (0.8, 1.7)	0.868
Radiologic findings			
Lobar consolidation	6 (26)	42 (48)	0.091
Centrilobular nodule	8 (35)	34 (39)	>0.999
GGO	8 (35)	12 (14)	0.030

Continuous variables were expressed as means ± SDs^a^ or medians (IQRs)^b^ and were compared by the Student's t test ^a^ or Mann-Whitney U test^b^

CURB-65: Confusion-Urea-Respiratory-Blood pressure-65 score, PSI: Pneumonia severity index, GGO: Ground-glass opacity.

### Independently associated factors and predictors of viral pneumonia in patients with CAP

Independently associated factors of viral pneumonia in patients with CAP are shown in Table 4. Rhinorrhea (OR 3.52; 95% CI, 1.58–7.87) and GGO (OR 4.68; 95% CI, 2.48–8.89) were seen as independent risk factors for viral pneumonia in patients with CAP.

**Table 4:** Independently associated factors for viral pneumonia in patients with community-acquired pneumonia.

Risk Factor	Logistic regression analysis without variable selection				Logistic regression analysis with backward conditional variable selection			
	OR	95% CI		*P* value	OR	95% CI		*P* value
		Lower	Upper			Lower	Upper	
Rhinorrhea	3.59	1.59	8.10	0.002	3.52	1.58	7.87	0.002
Lymphocyte %	1.01	0.98	1.04	0.436				
Creatinine	0.63	0.39	1.02	0.629				
GGO	4.35	2.27	8.34	<0.001	4.68	2.46	8.89	<0.001

GGO: ground glass opacity.

**Figure 1:**
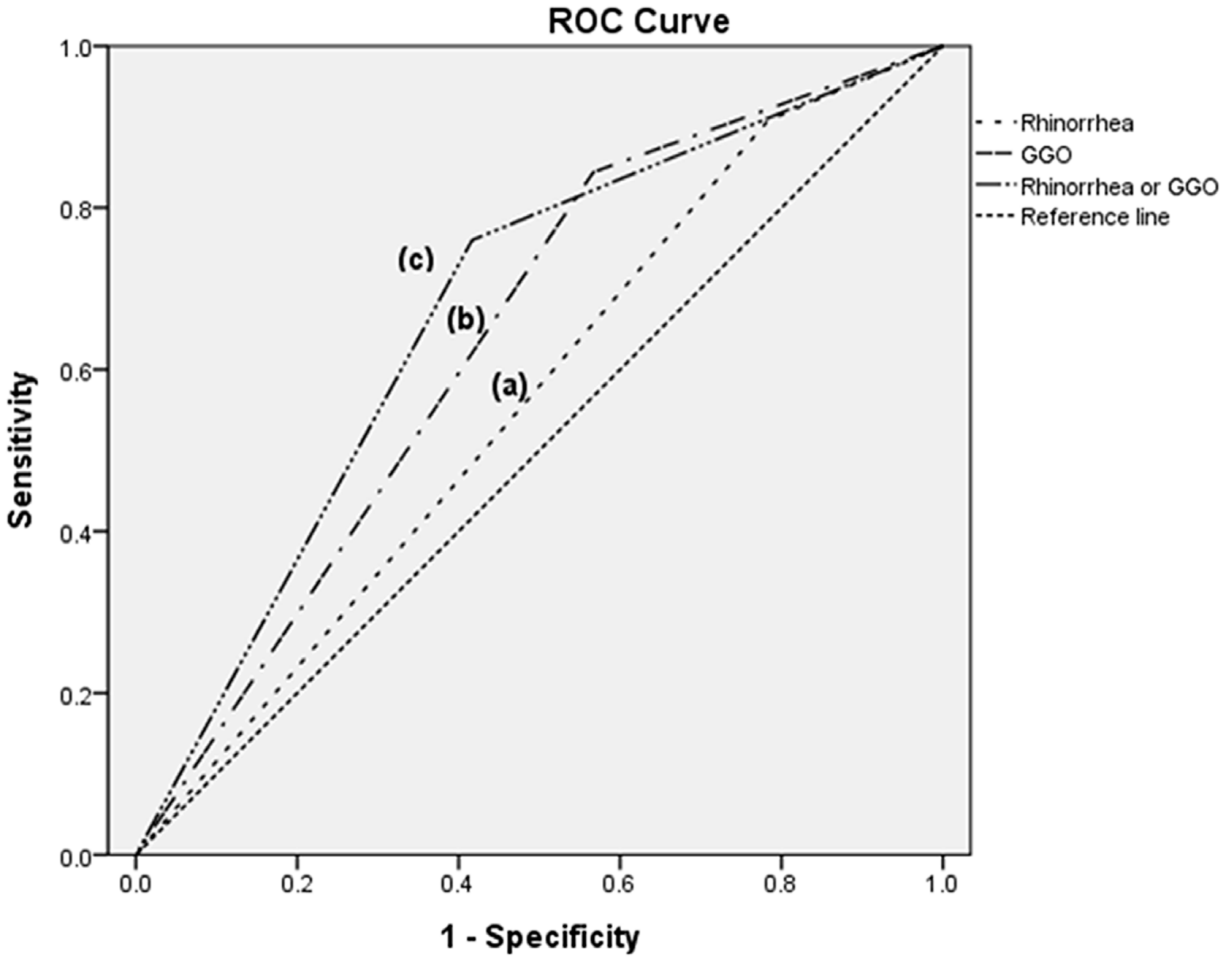
Receiver operating characteristics (ROC) curves of rhinorrhea and ground glass opacity in chest imaging as a predictor of viral pneumonia in 310 patients with community-acquired pneumonia. GGO: ground glass opacity. Area under the curve: (a) rhinorrhea, 0.562 (95% CI, 0.477–0.647); (b) GGO, 0.639 (95% CI, 0.555–0.723); (c) GGO or rhinorrhea, 0.672 (95% CI, 0.592–0.751).

The sensitivity, specificity, PPV and NPV of rhinorrhea were 22%, 91%, 36% and 83%, respectively. The sensitivity, specificity, PPV and NPV of GGO were and 43%, 84%, 40% and 86%, respectively. Fig. 1 shows the Receiver operating characteristics (ROC) curves for rhinorrhea, GGO and rhinorrhea or GGO for predicting viral pneumonia CAP. The resulting of Area under the curves (AUCs) were 0.562 (95% CI, 0.477–0.647) for rhinorrhea, 0.639 (95% CI, 0.555–0.723) for GGO, and 0.672 (95% CI, 0.592–0.751) for GGO or rhinorrhea.

Because the etiologic role of rhinoviruses, coronaviruses, and bocaviruses in patients with CAP is still in debate [30], [31], we analyzed the data after excluding patients who were tested using a PCR and shown to have rhinoviruses, coronaviruses, and bocaviruses, and it was found that the results were the same (S1 Table, S2 Table and S3 Table).

## Discussion

In this study, rhinorrhea and the GGO radiologic pattern were independently associated with viral pneumonia, and were specific predictors of viral pneumonia in patients with CAP.

The use of highly sensitive diagnostic tests in CAP patients increased the number of microbiological diagnoses and enabled identification of viral infection, despite an unknown etiology in ∼50% of cases [32]. It is estimated that 100 million cases of viral pneumonia occur each year [30]. The prevalence of viral infection was 22–33% in CAP, and influenza viruses accounted for most cases of viral pneumonia (6–8% of CAP) [7], [25], [32]. In this study, viral infection was detected in 18% of CAP, slightly lower than reported previously, but influenza pneumonia accounted for 7% of CAP, which is similar to previous reports. The slightly lower value for viral pneumonia is possibly due to a delay in applying the diagnostic tests for the virus because this study was performed across two referral centers, not primary care clinics.

Previous studies have demonstrated that the PSI score, ICU admission, need of mechanical ventilation and mortality rate were not different between bacterial and viral pneumonia [26]. Our results were consistent with previous findings, and no differences in severity of disease and mortality were observed between viral and bacterial CAP.

Previous studies showed some clinical parameters associated with viral pneumonia in patients with CAP, however, the results were inconsistent; age and an immunocompetent host were associated with viral pneumonia in some studies [33], [34], but not in others [25]. The presence of a cough was associated with a higher incidence of viral pneumonia in one study [25], but in another study, it was associated with a lower frequency of a dry cough [26]. Several studies showed that purulent sputum, high fever, chest pain, an altered mental state, and dyspnea were present in similar proportions to non-viral pneumonia [24]–[26]. In this study, none of the above parameters were associated with, or were predictive of, viral pneumonia. Only rhinorrhea was associated with a viral etiology.

Some studies showed that WBC and CRP were increased significantly in individuals with bacterial pneumonia, compared with viral pneumonia [4], [7], [35]; however, in another study, WBC count and CRP levels were not different between viral and non-viral CAP [26]. We found no differences in WBC count and CRP levels between viral and non-viral CAP, and these parameters were not helpful in differentiating the two. Univariate analysis showed that viral pneumonia was associated only with a significantly lower concentration of serum creatinine and a higher lymphocyte fraction than non-viral pneumonia; however, multivariate analysis revealed no differences in these parameters between viral and non-viral CAP. Serum creatinine level was also not associated with viral pneumonia.

Previous studies suggested that radiology imaging of viral pneumonia was not predictive of its origin, because both bacteria and viruses could induce a broad range of changes on radiographic images of the chest [7], [36], [37]. However, it was suggested that viral pneumonia should be considered when multifocal GGO findings are observed [38]. Previous studies did not evaluate the sensitivity, specificity, PPV and NPV of the radiology findings for viral pneumonia in patients with CAP, in terms of which parameters were of help in the clinical decision to commence antiviral treatment. In our study, GGO was not sufficiently sensitive to detect viral pneumonia (indicating that other radiology findings are also commonly present in viral pneumonia) but was highly specific for viral pneumonia in patients with CAP. In our study, an AUC of 0.672 may not be sufficient for a clinical decision. However, there are no other clinical parameters that are preferable or useful for making decisions concerning empirical antiviral agents in clinical practice, although such parameters are badly needed. In our study, 73 patients had rhinorrhea or GGO, but only 10 (14%) of those patients were treated with antiviral drugs empirically, while 25 (40%) of the remaining 63 patients with rhinorrhea or GGO were diagnosed with viral pneumonia and were not treated with empirical antiviral agents.

This study had several limitations. First, the etiologic microorganism was heterogeneous and not identified in 50% of patients with non-viral CAP. For this reason, Influenza pneumonia was compared, additionally, with only pneumococcal pneumonia which mostly accounts for CAP and similar results. Second, although this study was undertaken at two hospitals, they were referral centers, not primary health-care clinics. There is the possibility that patients with less severe CAP could have been included if data from a primary healthcare clinic had been available. Third, because of the retrospective study design, 28% of the CAP patients were not tested using the respiratory virus PCR test and were excluded from the study, although the medical doctors were educated and were in consensus regarding the routine prescription of the respiratory virus PCR for correct etiologic diagnosis of CAP patients at our hospital. Because the factors influencing the physicians' decision to prescribe respiratory virus PCR were not determined, they may have influenced our results as unmeasured confounding factors in the analysis. Fourth, we did not perform bronchoalveolar lavage for the diagnosis of viral pneumonia because its diagnostic role in viral pneumonia is still not well defined and review articles still recommend upper respiratory specimens for the diagnosis of viral pneumonia [7], [10], [32]. Fifth, serum procalcitonin levels, which indicate the acute phase of bacterial CAP, were not reviewed and analyzed in this study, because procalcitonin was not checked routinely in all patients. Further study is needed to evaluate the usefulness of novel biomarkers for predicting viral pneumonia in CAP, including procalcitonin. Sixth, there is a possibility of co-infection in patients with a positive viral PCR and no positive bacteriology because of the limited sensitivity of current diagnostic methods.

In conclusion, symptom of rhinorrhea and GGO on radiology findings were independently associated with viral pneumonia. The sensitivity of these parameters was low, which suggests that all patients with CAP should be tested for viral pneumonia. However, the high specificity of rhinorrhea and GGO suggests that these could be useful clinical indicators for empirical antiviral therapy such as oseltamivir, zanamivir, peramivir, and/or ribavirin for the patients with CAP [7], especially in severe or rapidly progressing cases.

## Supporting Information

S1 Table **Clinical features and outcomes of 295 patients with viral or non-viral community-acquired pneumonia.** Continuous variables were expressed as means ± SDs^a^ or medians (IQRs)^b^ and were compared by the Student's t test^a^ or Mann-Whitney U test^b^. CURB-65: Confusion-Urea-Respiratory-Blood pressure-65 score, PSI: Pneumonia severity index, ICU: Intensive care unit.
(DOCX)

S2 Table **Laboratory and radiological findings of 295 cases of viral or non-viral community-acquired pneumonia.** Continuous variables were expressed as medians (IQRs)^a^ and were compared by Mann-Whitney U test^a^. GGO: Ground-glass opacity.
(DOCX)

S3 Table **Independently associated factors for viral pneumonia in patients with community-acquired pneumonia.** GGO: ground glass opacity.
(DOCX)
