# Supplementary material for: Predictors of Viral Pneumonia in Patients with Community-Acquired Pneumonia
Source: PLoS One. 2014 Dec 22;9(12):e114710. doi: 10.1371/journal.pone.0114710 (PMC4273967; doi:10.1371/journal.pone.0114710)
Supplement: S1 Table — Clinical features and outcomes of 295 patients with viral or non-viral community-acquired pneumonia. Continuous variables were expressed as means ± SDsa or medians (IQRs)b and were compared by the Student's t testa or Mann-Whitney U testb. CURB-65: Confusion-Urea-Respiratory-Blood pressure-65 score, PSI: Pneumonia severity index, ICU: Intensive care unit. (DOCX) [file pone.0114710.s001.docx]

**Table S1. Clinical features and outcomes of 295 patients with viral or non-viral community-acquired pneumonia.**

| Characteristics | No. (%) of patients | | *P* value |
| --- | --- | --- | --- |
|  | Viral Pneumonia  (N=45) | Non-viral Pneumonia  (N=250) |  |
| Demographic data | | | |
| Male sex | 30 (67) | 194 (78) | 0.130 |
| Age ^a^ | 69 (±12) | 70 (±11) | 0.704 |
| Underlying diseases | | | |
| Diabetes mellitus | 10 (22) | 50 (20) | 0.692 |
| Hypertension | 12 (27) | 77 (31) | 0.724 |
| Cancer | 14 (31) | 45 (18) | 0.066 |
| Chronic obstructive lung disease | 9 (20) | 79 (32) | 0.156 |
| Ischemic heart disease | 5 (11) | 21 (8) | 0.568 |
| Cerebral vascular accident | 1 (2) | 14 (6) | 0.482 |
| Chronic kidney disease | 1 (2) | 13 (5) | 0.703 |
| Symptoms | | | |
| Fever | 32 (71) | 152 (61) | 0.242 |
| Cough | 33 (73) | 149 (60) | 0.653 |
| Sputum | 22 (49) | 134 (54) | 0.628 |
| Rhinorrhea | 10 (22) | 23 (9) | 0.018 |
| Dyspnea | 15 (33) | 112 (45) | 0.191 |
| Chest pain | 1 (2) | 26 (10) | 0.094 |
| Diarrhea | 3 (7) | 12 (5) | 0.710 |
| Severity and Outcomes | | | |
| CURB-65^b^ | 1.4 (1, 2) | 1.4 (1, 2) | 0.298 |
| PSI^b^ | 103 (71, 120) | 102 (73, 126) | 0.790 |
| ICU admission | 6 (13) | 44 (18) | 0.666 |
| Mechanical ventilation | 7 (16) | 30 (12) | 0.471 |
| 30-day mortality | 8/43 (19) | 38/249 (15) | 0.401 |
| 30-day attributable mortality | 8/43 (19) | 38/249 (15) | 0.401 |

Continuous variables were expressed as means ± SDs^a^ or medians (IQRs)^b^ and were compared by the Student’s t test ^a^ or Mann-Whitney U test^b^ .

CURB-65: Confusion-Urea-Respiratory-Blood pressure-65 score, PSI: Pneumonia severity index, ICU: Intensive care unit
