# Supplementary material for: Predictors of Viral Pneumonia in Patients with Community-Acquired Pneumonia
Source: PLoS One. 2014 Dec 22;9(12):e114710. doi: 10.1371/journal.pone.0114710 (PMC4273967; doi:10.1371/journal.pone.0114710)
Supplement: S2 Table — Laboratory and radiological findings of 295 cases of viral or non-viral community-acquired pneumonia. Continuous variables were expressed as medians (IQRs)a and were compared by Mann-Whitney U testa. GGO: Ground-glass opacity. (DOCX) [file pone.0114710.s002.docx]

**Table S2. Laboratory and radiological findings of 295 cases of viral or non-viral community-acquired pneumonia.**

| Characteristics | No. (%) of patients | | *P* value |
| --- | --- | --- | --- |
|  | Viral Pneumonia  (N=45) | Non-viral Pneumonia  (N=250) |  |
| Laboratory findings | | | |
| White blood cell counts (/mm^3^)^a^ | 12342 (7400, 16850) | 13946 (9950, 17120) | 0.103 |
| Neuotrophil %^a^ | 78 (73, 86) | 78 (76, 89) | 0.294 |
| Lymphocyte %^a^ | 14 (7, 18) | 11 (5, 14) | 0.023 |
| C-reactive protein (mg/dL)^a^ | 14 (6, 20) | 14 (5, 13) | 0.764 |
| Serum creatinine (mg/dL)^a^ | 1.1 (0.7, 1.4) | 1.4 (0.8, 1.5) | 0.141 |
| Radiologic findings | | | |
| Lobar consolidation | 13 (29) | 111 (44) | 0.070 |
| GGO | 18 (40) | 39 (16) | <0.001 |
| Centrilobular | 14 (31) | 100 (40) | 0.319 |

Continuous variables were expressed as medians (IQRs)^a^ and were compared by Mann-Whitney U test^a^.

GGO: Ground-glass opacity.
