# Supplementary material for: Predictors of Viral Pneumonia in Patients with Community-Acquired Pneumonia
Source: PLoS One. 2014 Dec 22;9(12):e114710. doi: 10.1371/journal.pone.0114710 (PMC4273967; doi:10.1371/journal.pone.0114710)
Supplement: S3 Table — Independently associated factors for viral pneumonia in patients with community-acquired pneumonia. GGO: ground glass opacity. (DOCX) [file pone.0114710.s003.docx]

**Table S3. Independently associated factors for viral pneumonia in patients with community-acquired pneumonia.**

| Risk Factor | | Logistic regression analysis  without variable selection | | | | | | | | Logistic regression analysis  with backward conditional variable selection | | | | | | | |
| --- | --- | --- | --- | --- | --- | --- | --- | --- | --- | --- | --- | --- | --- | --- | --- | --- | --- |
|  |  | OR | | 95 % CI | | | | *P* value | | OR | | 95 % CI | | | | *P* value | |
|  |  |  |  | Lower | | Upper | |  |  |  |  | Lower | | Upper | |  |  |
| Rhinorrhea | | 3.41 | | 1.43 | | 8.13 | | 0.006 | | 3.41 | | 1.43 | | 8.11 | | 0.005 | |
| Lymphocyte % | | 1.02 | | 0.99 | | 1.05 | | 0.207 | |  | |  | |  | |  | |
| Creatinine | | 0.73 | | 0.48 | | 1.13 | | 0.159 | |  | |  | |  | |  | |
| GGO | | 3.96 | | 1.94 | | 8.11 | | <0.001 | | 4.07 | | 2.00 | | 8.31 | | <0.001 | |

GGO: ground glass opacity.
